# Antioxidant Content and Antioxidant Capacity of the Protein-Rich Powdered Beverages Enriched with Flax Seeds Gum

**DOI:** 10.3390/antiox11030582

**Published:** 2022-03-18

**Authors:** Justyna Bochnak-Niedźwiecka, Urszula Szymanowska, Ireneusz Kapusta, Michał Świeca

**Affiliations:** 1 Department of Biochemistry and Food Chemistry, University of Life Sciences, Skromna Str. 8, 20-704 Lublin, Poland; urszula.szymanowska@up.lublin.pl; 2 Department of Food Technology and Human Nutrition, Rzeszów University, Zelwerowicza Str. 4, 35-601 Rzeszow, Poland; ikapusta@ur.edu.pl

**Keywords:** powdered beverages, flax seeds gum, antioxidant properties, nutrients digestibility, phenolics

## Abstract

Powdered beverages produced from dried fruit and vegetables are new products whose properties may be tailored by adding efficient nutrients and functional ingredients. The analyses of low-molecular antioxidants and antioxidant properties as well as nutrient content and digestibility were tested in beverages enriched with lentil proteins (AGF) and flaxseed gum (FSG). A replacement of sprouted lentil flour with the AGF deteriorated the phenolic content. As a main source of phenolics and vitamin C, lyophilized parsley leaves and broccoli sprouts were recognized. (There was no clear effect of the FGS.) The highest content of phenolics was determined in the beverages with these additives without the AGS (c.a. 125 μg/g). The AGF significantly improved the ability to quench ABTS radicals and reduce power. The best results were for the beverages without the FSG. (The effect was enhanced by lyophilized fruit and green vegetables.) The lowest chelating power and ability to quench hydroxyl radicals were in the beverages based on the AGF (improvement by the FSG and green vegetables). The tailoring of beverages’ recipes significantly increased protein content and did not affect nutrient digestibility. The modifications allow obtaining the beverages exhibiting multidirectional antioxidant properties, being a source of easily bioaccessible starch and proteins.

## 1. Introduction

Functional food products play an important role in modern nutrition. They not only provide basic nutrients but, due to a unique composition and resulting bioactivity, can also be an effective tool for improving quality of life. It is believed that they are an important element in the prevention of civilization diseases such as cancers, cardiovascular disease, or neurodegenerative disorders [1]. Meeting the high demands of consumers, food producers place great emphasis on expanding a wide range of so-called ready-to-eat products that are easy to store and prepare. An important branch of this market consists of products based on powdered vegetables and fruits. The water content of powdered food has to be at a low level; thus, different drying techniques (freeze-dried or convection-dried) are usually employed in their production. The pro-health properties of such products are associated with high contents of low-molecular antioxidants (phenolics, carotenoids), vitamins, and dietary fiber [2]. Additionally, a further improvement of both nutraceutical and nutritional quality may be reached by fortification or enrichment with functional components [3].

Powdered beverages are recognized as one of the most promising and challenging areas for technology development in the food processing sector [4]. These foods are simple to prepare and require being reconstituted before consumption [5]. Beverage formulation may be tailored by adding more efficient and nutrient-dense powders based on natural ingredients, such as prebiotics, sulforaphane, or phenolics [1]. Previously, new functional powdered beverages based on lyophilized lentil sprouts, carrots, and pumpkins have been designed [6,7]. Their organoleptic, nutritional, and pro-health properties were shaped by the addition of lyophilized broccoli sprouts, parsley leaves, strawberries, and raspberry fruits. The beverages are characterized by many functional health-promoting components such as starch, proteins, carotenoids, and phenolics [6,7]. Major factors negatively affecting organoleptic properties were the “cloudy” color and “beany” taste and odor coming from the sprouted flour and the low stability causing sedimentation of components. In current studies, sprouted lentil flour was replaced by lentil protein characterized by a desirable amino acids profile and high digestibility [8]. Lentil albumins and globulins are characterized by desirable technological properties, including foaming capacity and stability, emulsifying activity index, and water absorption [9]. Additionally, flaxseed gum (FSG), being an excellent source of soluble and insoluble dietary fiber [10], was introduced. This component is characterized by some desirable physical properties and exhibits many pro-health activities, e.g., improving water-holding capacity and solution stability, increasing anticancer and antioxidant properties, and acting as prebiotics [11,12,13,14].

The study aimed to evaluate the effects of beverage composition on its antioxidant capacity determined based on the test with a different mode of action. The modification of recipes includes the replacement of the sprouted lentil flour with the lentil proteins (AGF) as well as incorporation of flaxseed gum (FSG) and lyophilized antioxidant-rich vegetables (parsley leaves and broccoli sprout) and fruit (strawberry and raspberry) powders. Special attention was placed on the qualitative and quantitative analysis of low-molecular antioxidant content and the digestibility of main nutrients.

## 2. Materials and Methods

### 2.1. Chemicals

All chemicals and enzymes were purchased from Sigma-Aldrich Company (Poznan, Poland) and BTL Ltd. (Łodz, Poland).

### 2.2. Plant Material

All plant materials (carrot, pumpkin, lentil sprouts, lentil seeds, raspberry, strawberry, broccoli sprouts, parsley leaves, and defatted linseeds powder) were obtained from a local market. Before freeze-drying, the vegetables were peeled and grated to obtain ca. 2.5 mm pieces. The samples were lyophilized (LABCONCO, Kansas City, MO, USA) and milled using a laboratory grinder (MRC GRINDING MACHINE, SM-450, Holon, Israel) in 5 intervals (3 s) to avoid thermal degradation of active components. Finally, the powders were sieved (0.45 mm) and stored at −20 °C.

Flaxseeds gum (FSG) was prepared as described previously by Nikbakht Nasrabadi, Goli, Sedaghat Doost, and Van der Meeren [10]. The defatted powder was mixed with Milli-Q water with a ratio of 1:10 (*w*/*v*) and mixed at 200 rpm (80 °C, 2 h). Then, the mixture was passed through a screen (0.45 mm). Flaxseed gum (FSG) was precipitated by mixing with 96% ethanol (1:3, *w*/*v*), freeze-dried, milled using the laboratory grinder (MRC GRINDING MACHINE, SM-450, Holon, Israel), and stored at −20°C.

Lentil albumin/globulin-rich fraction (AGS) was isolated based on the solubility criterion according to Ribeiro, Teixeira, and Ferreira [15] with slight modifications. For the extraction of the AGF, flour (100 g) was extracted with 1 L of 10 mmol L^−1^ CaCl_2_, 10 mmol L^−1^ MgCl_2_, and 100 g kg^−1^ (*w*/*v*) NaCl in deionized water for 1 h at room temperature. The samples were centrifuged (15 min., 3860× *g*) and supernatants were collected. The solubilized proteins (AGF) were precipitated at 4 °C overnight by decreasing the pH value to 4.5 with 0.1 N HCl. After that, centrifugation at 3860× *g* for 10 min at 4 °C followed. The received AGF was resuspended in water, freeze-dried (LABCONCO, Kansas City, MO, USA), and stored at −20 °C.

### 2.3. Powdered Beverages Composition

The composition was designed based on the previously developed recipes for functional beverages. The output drinks contained 30% carrot, 30% pumpkin, and 40% lentil sprouts (C), 30% carrot, 30% pumpkin, 30% lentil sprouts, and 10% broccoli sprouts and parsley leaves (C1), and 30% carrot, 30% pumpkin, 30% lentil sprouts and 10% raspberry, strawberry (C2) [6,7]. The detailed composition of the studied beverages is presented in Table 1.

In the current study, the sprout lentil flour was replaced by the AGF. Additionally, functional flaxseeds gum (FSG) was added to the recipes (from 0 up to 15% (*w*/*w*)). Powdered beverages (0.5 g) were rehydrated in 10 mL Milli-Q water, shaken (3 intervals, 30 s) at room temperature using a multi-rotator (RS-60, Biosan, Otwock, Poland) (300 rpm), and used for analysis.

### 2.4. Phenolics Analysis

The beverages were mixed with 70 mL of acidified (pH 3) acetone–methanol (7:3 (*v*/*v*)) and further extracted for 15 min. at 30 °C using a significator (XUBA1, Grant Instruments, Cambridge, UK). Then, the samples were centrifuged (15 min, 6000× *g*) and organic solvents were removed using the Refrigerated CentriVap Concentrator (Labconco, Kansas City, MO, USA) for the UPLC analysis or stored at −20 °C for antioxidant tests.

The crude extract was passed through a C18 Sep-Pak (360 mg, 55–105 μm) cartridge (Waters Associates, Milford, MA, USA) preconditioned with water. The cartridge was washed first with water (10 mL) to remove sugars and then with MeOH (10 mL) to elute phenolics. This fraction was evaporated to dryness (Labconco, Kansas City, MO, USA) and redissolved in 50% MeOH for analyses. Structural information and general phenolic profiles were gathered using a Waters Acquity UPLC system consisting of a binary solvent manager, a sample manager, a PDA detector, and a triple quadrupole detector (TQD) Oszmiański, Wojdyło, Gorzelany, and Kapusta [16]. Polyphenolic compounds were analyzed using Waters ACQUITY system (Waters, Milford, MA, USA). The separation was carried out using a BEH C18 column (100 mm × 2.1 mm i.d., 1.7 µm, Waters) kept at 50 °C. For the anthocyanin investigation, the following solvent system: mobile phase A (2% formic acid in water *v*/*v*) and mobile phase B (2% formic acid in 40% ACN in water *v*/*v*) was applied. For other polyphenolic compounds, a lower concentration of formic acid was used (0.1% *v*/*v*). The gradient program was set as follows: 0 min 5% B, from 0 to 8 min linear to 100% B, and from 8 to 9.5 min for washing and back to initial conditions. The injection volume of the samples was 5 µL (partial loop with needle overfill) and the flow rate was 0.35 mL/min. The following parameters were used for TQD: capillary voltage 3.5 kV; con voltage 30 V in positive and negative mode; the source was kept at 250 °C and desolvation temperature was 350 °C; con gas flow 100 L/h; and desolvation gas flow 800 L/h. Argon was used as collision gas at a flow rate of 0.3 mL/min. Identification of individual compounds was completed based on the mass-to-charge ratio (*m*/*z*) recorded for negative and positive ions and on the basis of UV-Vis and fragmentation spectra of the MS/MS. Compounds were identified by comparing the obtained data with the literature data and available standards. Detailed information is presented in Table 2. Quantification was achieved by the injection of solutions of known concentrations ranging from 0.05 to 5 mg/mL (R2 ≤ 0.9998) of phenolic compounds as standards. All determinations were performed in triplicate and expressed as μg/g of powder. Waters MassLynx software v.4.1 was used for data acquisition and processing.

### 2.5. Vitamin C Content

Vitamin C content was determined according to the methods described by Mazurek and Jamroz [24]. The vitamin C from the beverages was extracted using 4.5% meta-phosphoric acid (MPA). The samples were centrifuged (15 min., 4 °C, 16,000× *g*) and used for analysis. Dehydroascorbic acid (DHA) was converted to ascorbic acid (AA) by using 5 mmol/L tris(2-carboxyethyl)phosphine. Analyses were performed with the Shimadzu UFLC system (Kyoto, Japan) equipped with a diode-array detector, isocratic pump, an autosampler, and a column thermostat. Separation was achieved at 30 °C using a column Gemini (150 × 4.6 mm, C18, Phenomenex, Torrance, CA, USA) connected with a pre-column Gemini (4 × 3 mm, C18, Phenomenex, USA) with orthophosphoric acid at pH 2.8 as a mobile phase. AA was identified based on the retention time and UV absorption and quantified using a calibration curve. Vitamin C content was expressed in μg per g of powder.

### 2.6. Carotenoids and Chlorophylls Content

The beverages were mixed with 90% acetone (final concentration of acetone 80%) and extracted until the powders became colorless. The samples were centrifuged (15 min, 6000× *g*) and the absorbance was measured at 663, 647, and 470 nm. The carotenoid and chlorophylls contents were calculated using equations proposed by Sumanta, Haque, Nishika, and Suprakash [25].

Ch_a_ = 12.25_A663_ − 279_A647_

Ch_b_ = 21.5_A647_ − 5.1_A663_

Carotenoids = (1000_A470_ − 1.82_Cha_ − 85.02_Chb_)/198

Carotenoids were expressed in μg per g of powder.

### 2.7. Antioxidant Properties

The beverages were mixed with 70 mL of acidified (pH 3) acetone–methanol (7:3 (*v*/*v*)) and further extracted. Finally, they were centrifuged (15 min, 6000× *g*) and used for analysis.

#### 2.7.1. Reducing Power (RP)

Reducing power was determined by the method of Pulido, Bravo, and Saura-Calixto [26]. Reducing power was expressed as Trolox equivalents (TE) in mg per g of powder or 100 mL of beverages.

#### 2.7.2. Ability to Quench ABTS Radicals

The experiments were carried out using the ABTS decolorization assay [27]. The free radical scavenging ability was expressed as Trolox equivalents in mg per g of powder or 100 mL of beverages.

#### 2.7.3. Ability to Quench Hydroxyl Radicals (•OH)

The •OH scavenging ability was determined according to Su, Wang, and Liu [28]. It was expressed as Trolox equivalents in mg per g of powder or 100 mL of beverages.

#### 2.7.4. Chelating Power

Chelating power was determined by the method of Decker and Welch [29]. Chelating power was expressed as EDTA equivalents in mg per g of powder or 100 mL of beverages.

### 2.8. Nutrients Analysis

#### 2.8.1. Protein Content

The beverage was mixed with 1 mol L^−1^ NaOH and extracted for 30 min. with 300 rpm (RS-60, Biosan). The samples were centrifuged (15 min, 6000× *g*) and protein content was determined using the Bradford method [30]. Total protein was expressed as albumin equivalents in mg per 100 mL of beverages.

#### 2.8.2. Free Amino Acids and Peptides

Non-protein nitrogen was determined with 2,4,6-trinitrobenzene sulfonic acid (TNBS) according to the methods described by Adler-Nissen [31] using L-leucine as the standard. Free amino acid and peptide content was expressed in mg per 100 mL of beverages.

#### 2.8.3. Starch Content

Starch content in the beverages was determined after hydrolysis with thermostable α-amylase and amyloglucosidase according to manufacture procedure (The Total Starch Kit, Megazyme, Auchincruive, UK). Total starch was expressed in mg per 100 mL of beverages.

#### 2.8.4. Free Reducing Sugars

For the free reducing sugar determination, the beverages were mixed with 50% methanol and extracted for 30 min. with 300 rpm (RS-60, Biosan). The samples were centrifuged at 12,000× *g* at 4 °C for 20 min, and the free reducing sugar content was determined by using the DNSA method [32]. Free reducing sugars were expressed in mg per 100 mL of beverages.

#### 2.8.5. Nutrients Digestibility

The in vitro digestibility of nutrients was evaluated according to the procedure described by Świeca and Baraniak [8]. In vitro digestion was performed according to the methodology described by Minekus et al. [33]. For digestion, 2 mL of beverage was subjected. After digestion, the samples were mixed with an equal volume of methanol to stop enzyme action. The samples were centrifuged at 12,000× *g* at 4 °C for 20 min. The pellets were washed again with methanol. Undigested starch was determined using the Total Starch Kit. (Megazyme). Undigested proteins were determined in both the pellets after solubilization with 0.1 mol L^−1^ NaOH and the supernatants using the Bradford method [30]. Nutrient digestibility was calculated as the ratio of undigestible starch/proteins to total starch/proteins using the equation:

Digestibility [%] = 100 − [(undigestible starch or proteins/total starch or proteins) × 100%](1)

### 2.9. Statistical Analysis

The distribution of the data was estimated using Shapiro–Wilks tests. A statistical significance was estimated by Tukey’s test for the data obtained from three independent samples of each extract in three parallel experiments (*n* = 9). The experimental data were shown as means ± S.D. Unless stated otherwise, the statistical tests were carried out at a significance level of *α* = 0.05. The statistical tests were performed using Statistica 13.1 software (StatSoft, Inc., Tulsa, OK, USA).

## 3. Results and Discussion

### 3.1. Low-Molecular Antioxidants

Qualitative–quantitative analysis of the phenolics fraction from the studied beverages is presented in Table 3. A replacement of lyophilized lentil sprouts with the lentil proteins (AGF) significantly deteriorated the phenolic content. In the case of the beverages without any additives, a decrease by c.a. 58% was determined. There was no clear effect of the flaxseed gum (FSG) addition on the phenolic content in the protein-rich beverages (P0-P15). The highest content of phenolics was determined in the beverages containing lyophilized vegetables, which was especially visible in those without and with 5% addition of the FSG—ca. 4.4-fold increase compared to the control (P0). In this case, a negative effect coming from the replacement of sprouted lentil flour was compensated by functional components. This increase resulted from high contents of sinapoyl glucoside, kaempferol 3-*O*-malonyl-glucoside-7-*O*-glucuronide, kaempferol 3-*O*-malonyl-rhamnoside-7-*O*-pentoside, and kaempferol 3-*O*-malonyl-rutinoside, being the main phenolics of broccoli sprouts and parsley leaves. The main phenolics in the beverages enriched with lyophilized fruit were anthocyanins; however, high contents of kaempferol 3-*O*-glucoside-rutinoside-7-*O*-rhamnoside were also determined. The presence of kaempferol glucosides was previously reported in parsley leaves [34]. High contents of flavonoids and chlorogenic acids in broccoli sprouts are also confirmed by Pérez-Balibrea, Moreno, and García-Viguera [35]. A constant level of kaempferol 3-*O*-glucoside-rutinoside-7-*O*-rhamnoside in the beverages based on the AGF may suggest that this compound comes from lentils. This compound is a dominant phenolic of lentils [36], accounting for up to 40% of total phenolics. Although in the current study the isolated lentil protein fractions (AGF) were introduced (instead of sprouted flour) into recipes, it was previously proved that phenolics may strongly interact with albumin and globulins, being an integral component of legumes protein isolates [37]. Such a phenomenon was previously observed during the study of the binding capacity of legume albumin and globulins with a different class of phenolics [38]. Importantly, the introduction of the FSG into vegetable-based beverages caused a significant, dose-dependent decrease in the total phenolics. The variation in this parameter is the result of many factors. A decrease may be due to a lower content of lyophilized vegetables (the main source of phenolics) in the recipes of beverages with an increasing level of FSG. It is also proved that phenolics interact with the FSG components, which decreases their extractability by forming insoluble complexes. At pH 3 (extraction conditions), the FSG is negatively charged [10], which promotes a strong binding with flavonoid glucosides (additional hydroxylic groups support the strength of interaction). It was confirmed by Guimarães et al. [39] that at pH 4.5, nearly 32% and 38% of chlorogenic acid and phloridzin, respectively, were retained by debranched arabinan. On the other hand, such behavior was not visible in the case of fruit-based beverages. Anthocyanins complex with pectin and arabinoxylans [40] decreased their release into the extraction medium; however, mainly hydrogen bonds are employed in those interactions. On the other hand, that class of phenolics may be effectively released from the matrix during extraction under acidic conditions, supported by ultrasounds Nikbakht Nasrabadi et al. [10].

The improved extractability could be also explained by anthocyanin self-association facilitating the saturation transfer between anthocyanin molecules. Self-association is a very well-known phenomenon driven by hydrophobic vertical stacking between the anthocyanins to form π–π complexes [41].

The content of ascorbic acid in the beverages was tailored by the addition of lyophilized vegetables and fruit, as well as an incorporation of AGS and FSG (Table 4). The highest content of vitamin C was recorded in the beverages enriched with lyophilized parsley leaves and broccoli sprouts (ca. 1.3 mg/g). Both materials, except cultivar used or cultivation conditions, are excellent sources of ascorbic acid [2]. The replacement of sprouted lentil flour with the AGF caused a slight decrease in ascorbic acid level (by 13%), which directly comes from a lower content of components being a source of this antioxidant.

The incorporation of FSG into recipes caused a further decrease in the vitamin C content, which was due to a further lowering in the content of the functional incident. Ascorbic acid ranged from 71% to 85 % of total vitamin C; however, a clear trend was recorded—anthocyanins seem to protect the oxidation of these compounds. Similar behavior was previously recorded by Brenes, del Pozo-Insfran, and Talcott [42] in the model grape juice system containing anthocyanins and ascorbic acid. As expected, the addition of green vegetable powder increased the chlorophyll content (especially chlorophyll *a*). Its content in the beverage enriched with the lyophilized parsley and broccoli sprouts (P0V) was higher by 62% compared to the P0. The carotenoid content in the beverages based on the AGF was mainly tailored by the amount of lyophilized carrot and pumpkin and ranged from 219 μg/g (P15) to 276 μg/g (P15V).

### 3.2. Antioxidant Potential

**Figure 1:**
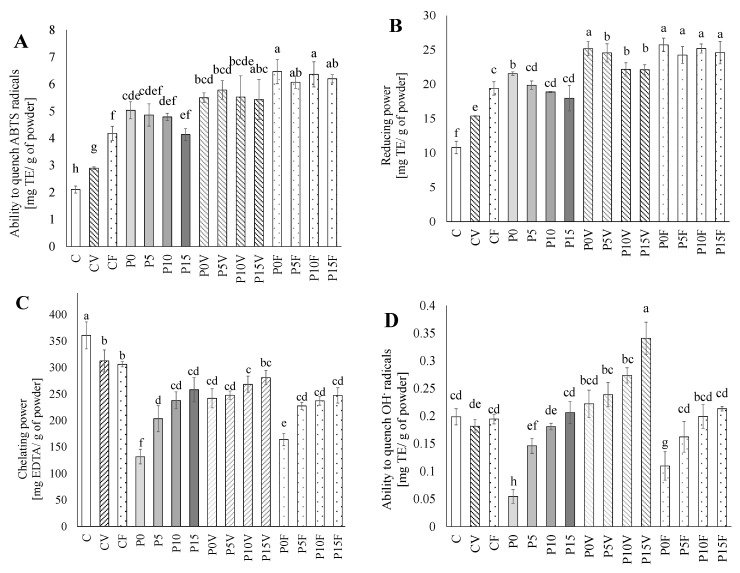
Antioxidant properties of powdered beverages. (**A**)—Ability to quench ABTS radicals; (**B**)—Reducing power; (**C**)—Chelating power; (**D**)—Ability to quench OH-radicals. C—beverages based on lentil sprouts; P—beverages based on lentil proteins; V—lyophilized parsley and broccoli sprouts; F—lyophilized strawberry and raspberry; 0–15—percent of flaxseeds gum. Means in each figure (A, B, C, D) (±SD) followed by different letters are significantly different (*n* = 9; *p* ≤ 0.05).

The replacement of sprouted lentil flour with AGF significantly improved the ability to quench ABTS radicals (Figure 1A) (1.2-fold increase). On the other hand, a slightly negative effect was observed after the introduction of the FSG. This deterioration was compensated by the addition of lyophilized fruit and beverages—a decrease caused by a linseed gum (FSG) was overlapped by the high antiradical properties of the introduced phenolics and vitamin C.

Similar behavior was observed in the case of reducing power. Only in the beverages with higher contents of the FSG enriched with the lyophilized vegetables was this activity slightly lower—by 13% when compared to the vegetable-based beverages without the linseed carbohydrates (Figure 1B). The result of the ability to scavenge ABTS radicals and reducing power are in opposition to those presented by Bouaziz et al. [13], who confirm the relatively high antiradical properties of flax polysaccharides that are associated with a residual presence of phenolics. The contents of phenolics in the beverages based on the AGF suggest that the FSG did not contain any significant amounts of phenolics (Table 3). The increase in the mentioned abilities may be caused by the AGF components, especially kaempferol 3-*O*-glucoside-rutinoside-7-*O*-rhamnoside. A level of the activities in the beverages enriched with the lyophilized vegetables and fruit may be partially explained by the interaction of antioxidants with the beverage matrix, especially phenolic, protein and polysaccharides. The presence of such complexes significantly reduces their functionality. It has been shown that phenolics can effectively bind to the legume proteins, which caused a significant reduction in the amount of free functional groups responsible for antioxidant properties [38]. The highest chelating power was determined in the control beverages based on the sprouted lentil flour (Figure 1C). The observed decrease was probably caused by a reduction in kaempferol 3-*O*-glucoside-rutinoside-7-*O*-rhamnoside content observed after the AGF addition. Although the beverages based on the AGF (P0) are characterized by the lowest chelating power, their undesirable characteristic was successfully improved by the incorporation of FSG as well as lyophilized vegetables. An addition of 15% FSG duplicated the chelating power. The obtained results are confirmed by previous studies concerning the chelating power of exopolysaccharides from soybean [43] and acidic polysaccharide fraction from pink oysters [44]. A key role of functional groups such as carboxyl group and sulfuric radicals in the structure of the fractionated four polysaccharides in improving this activity was also confirmed Fan et al. [45]. The positive effect created by the linseed polysaccharides (FSG) was supported by the phenolics fraction from vegetables. It was previously reported that the incorporation of powdered broccoli sprouts [46] and parsley leaves [47] significantly improves the antioxidant properties (including the chelating power) of the fortified bread and pasta. Surprisingly, the addition of lyophilized fruit into the beverage based on the AGF improved its chelating properties, but after further incorporation of FSG (characterized by a high ability to sequestrate metal ions-clary visible in the beverages P0–P15, Figure 1C), it did not cause any additional changes. It seems that the FSG and anthocyanins act antagonistically. It may be suggested that anthocyanins interact with linseed polysaccharides or proteins, limiting the binding sides responsible for the chelation of the transition metal ions. Such behavior was previously observed with pectin and pectin fragments in the model solutions [48] and soybean proteins [49]. A similar pattern of activity was determined for the ability to quench the hydroxyl radicals; however, in this case, a positive impact of lyophilized vegetables was clearly visible (Figure 1D). Such behavior may suggest a potential mechanism of this activity, including the sequestration of iron ions responsible for generating •OH radicals. What is important is that the beverages with the highest content of the FSG and powdered vegetables exhibited activity higher by 71% compared to the beverages based on the sprouted lentil flour.

### 3.3. Nutrients Analysis

**Figure 2:**
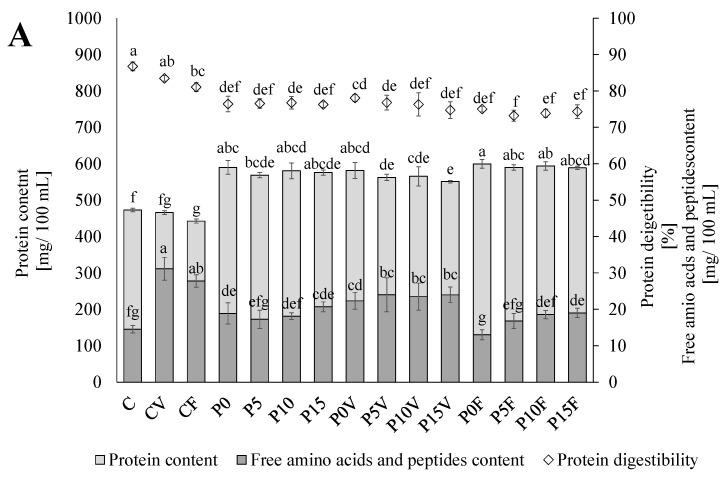

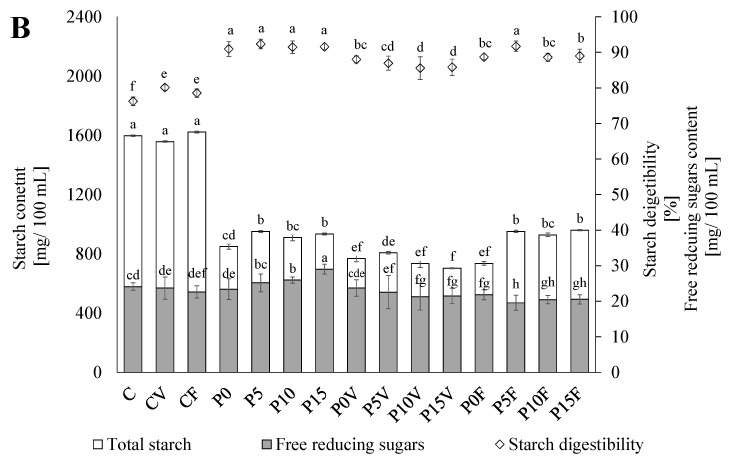
Protein (**A**) and starch (**B**) content and digestibility. C—beverages based on lentil sprouts; P—beverages based on lentil proteins; V—beverages enriched with the lyophilized parsley and broccoli sprouts; F—beverages enriched with the lyophilized strawberry and raspberry; 0–15—percent of flaxseeds gum added. Means in the columns (±SD) followed by different letters are significantly different (*n* = 9; *p* ≤ 0.05).

As expected, the replacement of the sprouted lentil flour with the AGF significantly increased protein content in the beverages (Figure 2A). An addition of lyophilized fruit and vegetables did not cause any significant changes in the total protein content and digestibility.

Similar observations were recorded in a study describing an effect of anthocyanins on the digestibility of soybean proteins where, despite an initial lowering of the protein accessibility for hydrolysis, finally there were no negative effects [49]. An insignificant negative influence on protein digestibility was observed in the beverages enriched with the FSG. Despite this, the digestibility of proteins was high (ranged from 85% to 92%) and corresponds well with that previously reported by Ghumman et al. [9]. An opposite relationship was observed in the case of starch. The content was significantly decreased, but its digestibility was high and was only slightly affected by the addition of lyophilized fruits and vegetables as well as the FSG. The lower digestibility of starch in the beverages enriched with phenolic-rich materials (lyophilized fruit and vegetables) may be due to the inhibition of amylases, which was previously reported in the case of *Rumex maderensis* phenolics [50] or anthocyanins from pigmented rice [51]. It may be also suggested that a decrease was caused by the formation of poorly soluble complexes, which was previously observed for the mixture of bean albumin and globulin with selected phenolic acids and flavonoids [38]. Compared to the beverages based on the sprouted lentil flour, the starch digestibility was increased by ca. 12–20%. It may be suggested that this is due to a lower content of condensed tannin commonly present in seed coats, which usually modifies both starch accessibility and hydrolases activities [52,53]. A slight negative impact on this parameter was observed after the incorporation of the FSG into the beverages enriched with powdered vegetables; however, a decrease did not exceed 3%. The addition of the FSG also increased the free sugar content, which was especially visible in the beverages without the powdered vegetables and fruit. It was previously proved that this function ingredient is a rich source of free sugars, including xylose, galactose, and rhamnose [14].

## 4. Conclusions

The applied modifications of beverage recipes allow obtaining products exhibiting multidirectional antioxidant properties. Compared to the counterparts based on the sprouted lentil flours without the prebiotic flaxseed gum, new beverages usually contained lower amounts of phenolics and carotenoids. Despite this fact, they were characterized by significantly higher reducing power and the ability to quench synthetic radicals. It seems that a crucial role, in this case, was played by bioactive components coming from the lyophilized fruit and green vegetables (mainly phenolics). The flaxseed gum turned out to be responsible for the elevation of chelating properties and the ability to scavenge physiological radicals. These properties were additionally improved by the addition of lyophilized parsley leaves and broccoli sprouts. What is important is that newly developed products are a source of easily bioaccessible starch and proteins. In sum, it may be concluded that the studied beverages are characterized by high functionality created by both ingredients and their interactions.

## Figures and Tables

**Table 1:** Composition of the powdered beverages.

Beverage	Lyophilized Components [%]						
	Sprouted Lentil Flour	Lentil Protein (AGF)	Carrot	Pumpkin	Vegetables	Fruit	Flaxseed Gum (FSG)
C	40	0	30	30	0	0	0
CV	30	0	30	30	10	0	0
CF	30	0	30	30	0	10	0
P0	0	40	30	30	0	0	0
P5	0	37	29	29	0	0	5
P10	0	36	27	27	0	0	10
P15	0	35	25	25	0	0	15
P0V	0	30	30	30	10	0	0
P5V	0	28	29	29	9	0	5
P10V	0	28	27	27	8	0	10
P15V	0	26	26	26	7	0	15
P0F	0	30	30	30	0	10	0
P5F	0	28	29	29	0	9	5
P10F	0	28	27	27	0	8	10
P15F	0	26	26	26	0	7	15

C—control beverages based on lentil sprouts; CV—beverages based on lentil sprouts enriched with lyophilized vegetables; CF—beverages based on lentil sprouts enriched with lyophilized fruit; P0-P15—beverages based on lentil proteins enriched with a flaxseed gum (FSG); P0V-P15V—beverages based on lentil proteins enriched with a flaxseed gum (FSG) and lyophilized vegetables; P0F-P15F—beverages based on lentil proteins enriched with a flaxseed gum (FSG) and lyophilized fruit.

**Table 2:** Phenolics identification.

	Compound	RT	Ion +/−	Fragment Ions	Absorbance Maxima	Refrences
		(min.)	(*m*/*z*)	(*m*/*z*)	(nm)	
	Anthocyanins					
A1	Cyanidin 3-*O*-diglucoside	2.47	611 ^+^	287	279, 515	S *
A2	Cyanidin 3-*O*-caffeoyl-rutinoside	2.55	757 ^+^	611, 287	279, 515	[17]
A3	Cyanidin 3-*O*-glucoside	2.69	449 ^+^	287	279,515	S
A4	Cyanidin 3-*O*-rutinoside	2.80	595 ^+^	287	278, 515	S
A5	Pelargonidin 3-*O*-glucoside	3.08	433 ^+^	271	276, 502	S
A6	Pelargonidin 3-*O*-malonyl-glucoside	3.77	519 ^+^	433, 271	269, 502	[18]
	Other phenolics					
P1	Kaempferol 3-*O*-glucoside-rutinoside-7-*O*-rhamnoside	3.19	901 ^−^	755, 285	265, 345	[19]
P2	Chlorogenic acid	3.32	353 ^−^	191	299 sh, 329	S
P3	Sinapoyl glucoside	3.39	385	223	299 sh, 329	[20]
P4	Kaempferol 3-*O*-rutinoside-7-*O*-glucoside	3.66	725	563, 285	263, 349	[19]
P5	Kaempferol 3-*O*-rhamnoside-7-*O*-pentoside	5.11	563	431, 285	266, 336	[19]
P6	Kaempferol 3-*O*-rutinoside	5.43	593	285	266, 336	S
P7	Kaempferol 3-*O*-malonyl-glucoside-7-*O*-glucuronide	5.61	709	623, 461, 285	266, 336	[21]
P8	Kaempferol 3-*O*-malonyl-rhamnoside-7-*O*-pentoside	5.73	649	563, 431, 285	266, 336	[22]
P9	Kaempferol 3-*O*-acetyl-rutinoside	5.89	635	593, 285	266, 345	[22]
P10	Kaempferol 3-*O*-malonyl-rutinoside	5.97	679	593, 285	266, 345	[22]
P11	Caffeoyl pinoresinol	6.15	591	357	299 sh, 329	[23]
P12	Undefined caffeoyl derivative	6.32	960	579	299 sh, 326	-

* S—identification based on comparison with authentic standards.

**Table 3:** Phenolic profile and content (μg/g of powder) of powdered beverages.

	C	CV	CF	P0	P5	P10	P15	P0V	P5V	P10V	P15V	P0F	P5F	P10F	P15F
A1	nd.	nd.	6.0 ± 0.8 ^a^	nd.	nd.	nd.	nd.	nd.	nd.	nd.	nd.	3.8 ± 0.5 ^c^	4.0 ± 05 ^c^	4.3 ± 0. 6^bc^	4.9 ± 0.6 ^b^
A2	nd.	nd.	3.0 ± 0.7 ^a^	nd.	nd.	nd.	nd.	nd.	nd.	nd.	nd.	2.3 ± 0.5 ^a^	2.4 ± 0.5 ^a^	2.4 ± 0.6 ^a^	2.6 ± 0.6 ^a^
A3	nd.	nd.	2.9 ± 0.4 ^a^	nd.	nd.	nd.	nd.	nd.	nd.	nd.	nd.	2.4 ± 0.3 ^b^	2.4 ± 0.3 ^b^	2.5 ± 0.3 ^b^	2.5 ± 0.3 ^ab^
A4	nd.	nd.	2.5 ± 0.1 ^a^	nd.	nd.	nd.	nd.	nd.	nd.	nd.	nd.	2.2 ± 0.1 ^d^	2.2 ± 0.1 ^d^	2.3 ± 0.1 ^c^	2.4 ± 0.1 ^b^
A5	nd.	nd.	9.2 ± 1.5 ^a^	nd.	nd.	nd.	nd.	nd.	nd.	nd.	nd.	5.9 ± 1.0 ^c^	6.8 ± 1.1 ^bc^	6.8 ± 1.1 ^bc^	8.1 ± 1.3 ^ab^
A6	nd.	nd.	2.9 ± 0.4 ^a^	nd.	nd.	nd.	nd.	nd.	nd.	nd.	nd.	2.4 ± 0.3 ^b^	2.6 ± 0.4 ^ab^	2.6 ± 0.4 ^ab^	2.8 ± 0.4 ^ab^
P1	26.2 ± 0.7 ^a^	18.2 ± 0.5 ^c^	20.2 ± 0.6 ^b^	8.0 ± 0.2 ^d^	6.0 ± 0.2 ^ef^	6.2 ± 0.2 ^ef^	5.9 ± 0.2 ^fg^	5.3 ± 0.2 ^g^	5.6 ± 0.2 ^fg^	3.3 ± 0.1 ^h^	3.8 ± 0.1 ^h^	7.9 ± 0.2 ^d^	6.2 ± 0.2 ^ef^	5.7 ± 0.2 ^fg^	6.6 ± 0.2 ^e^
P2	0.2 ± 0.1 ^o^	4.3 ± 0.1 ^d^	0.3 ± 0.1 ^n^	0.9 ± 0.1 ^g^	0.6 ± 0.1 ^j^	0.5 ± 0.1 ^l^	0.7 ± 0.1 ^h^	6.8 ± 0.1 ^a^	6.0 ± 0.1 ^b^	4.5 ± 0.1 ^c^	1.4 ± 0.1 ^e^	0.5 ± 0.1 ^k^	0.4 ± 0.1 ^m^	0.7 ± 0.1 ^i^	1.1 ± 0.1 ^f^
P3	0.3 ± 0.1 ^g^	14.7 ± 0.3 ^c^	1.0 ± 0.1 ^f^	0.2 ± 0.1 ^g^	0.1 ± 0.1 ^g^	0.2 ± 0.1 ^g^	0.2 ± 0.1 ^g^	19.9 ± 0.4 ^a^	16.5 ± 0.4 ^b^	12.4 ± 0.3 ^d^	6.1 ± 0.1 ^e^	1.4 ± 0.1 ^ef^	1.0 ± 0.1 ^f^	1.3 ± 0.1 ^ef^	1.7 ± 0.1 ^f^
P4	0.3 ± 0.1 ^f^	nd.	0.6 ± 0.1 ^cd^	0.2 ± 0.1 ^gh^	0.3 ± 0.1 ^f^	0.3 ± 0.1 ^f^	0.2 ± 0.1 ^fg^	1.7 ± 0.1 ^a^	1.3 ± 0.1 ^b^	0.5 ± 0.1 ^e^	0.3 ± 0.1 ^fg^	0.2 ± 0.1 ^h^	0.6 ± 0.1 ^d^	0.5 ± 0.1 ^e^	0.7 ± 0.1 ^c^
P5	0.3 ± 0.1^i^	5.3 ± 0.2 ^d^	1.5 ± 0.1 ^fg^	0.3 ± 0.1 ^i^	0.2 ± 0.1 ^i^	0.1 ± 0.1 ^i^	0.1 ± 0.1 ^i^	8.6 ± 0.4 ^b^	11.2 ± 0.5 ^a^	6.7 ± 03 ^c^	3.3 ± 0.1 ^e^	1.1 ± 0.1 ^h^	1.1 ± 0.1 ^h^	1.4 ± 0.1 ^gh^	1.8 ± 0.1 ^f^
P6	0.2 ± 0.1 ^i^	2.5 ± 0.1 ^c^	0.3 ± 0.1 ^hi^	0.7 ± 0.1 ^de^	0.7 ± 0.1 ^d^	0.5 ± 0.1 ^defgh^	0.5 ± 0.1 ^defg^	3.5 ± 0.2 ^b^	4.5 ± 0.3 ^a^	2.7 ± 0.2 ^c^	2.7 ± 0.2 ^c^	0.6 ± 0.0 ^def^	0.5 ± 0.1 ^efgh^	0.4 ± 0.1 ^fgh^	0.4 ± 0.1 ^ghi^
P7	0.1 ± 0.1 ^f^	44.8 ± 3.3 ^a^	nd.	0.9 ± 0.1 ^f^	0.8 ± 0.1 ^f^	0.5 ± 0.1 ^f^	2.9 ± 0.2 ^e^	12.8 ± 0.9 ^b^	12.0 ± 0.9 ^b^	8.3 ± 0.6 ^c^	5.0 ± 0.4 ^d^	0.7 ± 0.1 ^f^	0.3 ± 0.1 ^f^	0.3 ± 0.1^f^	0.3 ± 0.1 ^f^
P8	nd.	nd.	nd.	nd.	0.8 ± 0.1 ^e^	nd.	nd.	35.3 ± 0.6 ^b^	37.7 ± 0.7 ^a^	22.4 ± 0.4 ^c^	21.3 ± 0.4 ^d^	nd.	0.6 ± 0.1 ^e^	nd.	nd.
P9	nd.	nd.	nd.	nd.	0.2 ± 0.1 ^f^	nd.	nd.	1.6 ± 0.1 ^b^	2.0 ± 0.1 ^a^	0.9 ± 0.1 ^c^	0.9 ± 0.1 ^d^	0.3 ± 0.1 ^e^	0.2 ± 0.1 ^f^	0.3 ± 0.1 ^ef^	0.2 ± 0.1 ^g^
P10	0.4 ± 0.1 ^f^	12.5 ± 0.3 ^a^	nd.	0.3 ± 0.1 ^fg^	0.1 ± 0.1 ^fgh^	0.3 ± 0.1 ^fg^	0.3 ± 0.1 ^f^	10.4 ± 0.2 ^c^	11.4 ± 0.2 ^b^	6.4 ± 0.1 ^d^	6.1 ± 0.1 ^e^	0.3 ± 0.1 ^fg^	0.1 ± 0.1 ^gh^	0.3 ± 0.1 ^fg^	0.3 ± 0.1 ^fg^
P11	0.3 ± 0.1 ^f^	3.0 ± 0.1 ^c^	nd.	0.2 ± 0.1 ^i^	0.3 ± 0.1 ^ghi^	0.3 ± 0.1 ^fg^	0.3 ± 0.1 ^fg^	3.7 ± 0.1 ^a^	3.3 ± 0.1 ^b^	2.2 ± 0.1 ^d^	1.0 ± 0.1 ^e^	0.2 ± 0.1 ^hi^	0.3 ± 0.1 ^fgh^	0.3 ± 0.1 ^ghi^	0.3 ± 0.1 ^fg^
P12	0.3 ± 0.1 ^d^	13.5 ± 1.7 ^a^	nd.	0.3 ± 0.1 ^d^	0.3 ± 0.1 ^d^	0.2 ± 0.1 ^d^	0.3 ± 0.1 ^d^	14.6 ± 1.8 ^a^	13.2 ± 1.6 ^a^	9.8 ± 1.2 ^b^	5.4 ± 0.1 ^c^	0.2 ± 0.1 ^d^	0.1 ± 0.1 ^d^	0.3 ± 0.1 ^d^	0.2 ± 0.1 ^d^
TOTAL	28.55	118.81	50.54	11.97	10.25	9.02	11.57	124.10	124.64	80.10	57.11	32.55	31.77	32.37	36.71

A1—cyanidin 3-*O*-diglucoside; A2—cyanidin 3-*O*-caffeoyl-rutinoside; A3—cyanidin 3-*O*-glucoside; A4cyanidin 3-*O*-rutinoside; A5—pelargonidin 3-*O*-glucoside; A6—pelargonidin 3-*O*-malonyl-glucoside; P1—kaempferol 3-*O*-glucoside-rutinoside-7-*O*-rhamnoside; P2—chlorogenic acid; P3—sinapoyl glucoside; P4—kaempferol 3-O-rutinoside-7-O-glucoside; P5—kaempferol 3-*O*-rhamnoside-7-*O*-pentoside; P6—kaempferol 3-*O*-rutinoside; P7—kaempferol 3-*O*-malonyl-glucoside-7-*O*-glucuronide; P8—kaempferol-3-*O*-malonyl-rhamnoside-7-*O*-pentoside; P9—kaempferol 3-*O*-acetyl-rutinoside; P10—kaempferol 3-*O*-malonyl-rutinoside; P11—caffeoyl pinoresinol; P12—undefined caffeoyl derivative. nd.—not detected. C—beverages based on lentil sprouts; P—beverages based on lentil proteins; V—lyophilized parsley and broccoli sprouts; F—lyophilized strawberry and raspberry; 0–15—percent of flaxseeds gum. Means in the raws (±SD) followed by different letters are significantly different (*n* = 9; *p* ≤ 0.05).

**Table 4:** Content of vitamin C, carotenoids, and chlorophylls in powdered beverages.

	Ascorbic Acid [μg/g of Powder]	Dehydroascorbic Acid [μg/g of Powder]	Vitamin C [μg/g of Powder]	Chlorophyll a [μg/g of Powder]	Chlorophyll b [μg/g of Powder]	Carotenoids [μg/g of Powder]
C	468 ± 9.6 ^g^	145 ± 11.2 ^d^	613 ± 20.8 ^g^	98.7 ± 0.4 ^d^	90.4 ± 1.5 ^b^	255 ± 8.3 ^abc^
CV	1105 ± 30.4 ^a^	253 ± 6.8 ^bc^	1358 ± 23.7 ^a^	158 ± 3.5 ^ab^	98.7 ± 0.9 ^a^	250 ± 7.4 ^abc^
CF	918 ± 9.8 ^d^	121 ± 6.6 ^d^	1039 ± 16.4 ^e^	99.5 ± 1.3 ^d^	89.6 ± 0.3 ^b^	190 ± 8.9 ^d^
P0	412 ± 0.1 ^h^	162 ± 5.8 ^d^	573 ± 5.9 ^gh^	100 ± 2.5 ^d^	92.1 ± 2.9 ^b^	254 ± 20.4 ^abc^
P5	416 ± 2.8 ^h^	161 ± 2.5 ^d^	577 ± 0.3 ^gh^	103 ± 6.8 ^d^	91.0 ± 1.2 ^b^	230 ± 8.6 ^bc^
P10	407 ± 2.0 ^h^	121 ± 9.8 ^d^	528 ± 7.8 ^hi^	101 ± 2.4 ^d^	90.4 ± 1.1 ^b^	230 ± 4.4 ^bc^
P15	356 ± 31.6 ^i^	125 ± 41.9 ^d^	481 ± 73.5 ^i^	99.3 ± 1.4 ^d^	90.2 ± 1.1 ^b^	219 ± 8.0 ^cd^
P0V	1014 ± 4.1 ^b^	310 ± 27.7 ^a^	1324 ± 23.7 ^ab^	162 ± 6.2 ^a^	101.4 ± 2.0 ^a^	274 ± 12.6 ^a^
P5V	957 ± 12.1 ^c^	302 ± 19.1 ^a^	1259 ± 31.2 ^b^	156 ± 5.8 ^ab^	101 ± 2.3 ^a^	271 ± 30.9 ^a^
P10V	914 ± 11.1 ^d^	268 ± 29.3 ^ab^	1181 ± 18.2 ^c^	152 ± 2.8 ^bc^	99.2 ± 1.2 ^a^	276 ± 6.6 ^a^
P15V	894 ± 5.8 ^d^	214 ± 33.7 ^c^	1107 ± 39.5 ^d^	147 ± 1.2 ^c^	99.1 ± 2.2 ^a^	276 ± 19.4 ^a^
P0F	796 ± 5.5 ^e^	138 ± 4.4 ^d^	934 ± 1.1 ^f^	98.9 ± 0.5 ^d^	89.6 ± 0.5 ^b^	275 ± 17.9 ^a^
P5F	781 ± 11.2 ^e^	150 ± 7.0 ^d^	931 ± 4.2 ^f^	98.7 ± 2.4 ^d^	91.0 ± 2.2 ^b^	274 ± 10.1 ^a^
P10F	740 ± 12.0 ^f^	148 ± 0.4 ^d^	888 ± 12.4 ^f^	99.5 ± 1.3 ^d^	90.2 ± 1.1 ^b^	272 ± 8.9 ^a^
P15F	736 ± 6.6 ^f^	154 ± 5.2 ^d^	891 ± 11.7 ^f^	99.5 ± 1.3 ^d^	89.6 ± 0.9 ^b^	258 ± 23.1 ^ab^

C—beverages based on lentil sprouts; P—beverages based on lentil proteins; V—beverages enriched with the lyophilized parsley and broccoli sprouts; F—beverages enriched with the lyophilized strawberry and raspberry; 0–15—percent of flaxseeds gum added. Means in the columns (±SD) followed by different letters are significantly different (*n* = 9; *p* ≤ 0.05).

## Data Availability

Not applicable.
